# LINKS: Scalable, alignment-free scaffolding of draft genomes with long reads

**DOI:** 10.1186/s13742-015-0076-3

**Published:** 2015-08-04

**Authors:** René L. Warren, Chen Yang, Benjamin P. Vandervalk, Bahar Behsaz, Albert Lagman, Steven J. M. Jones, Inanç Birol

**Affiliations:** BC Cancer Agency, Michael Smith Genome Sciences Centre, Vancouver, British Columbia V5Z 4S6 Canada

**Keywords:** Nanopore sequencing, Scaffolding, Genome assembly, Next-generation sequencing, LINKS

## Abstract

**Background:**

Owing to the complexity of the assembly problem, we do not yet have complete genome sequences. The difficulty in assembling reads into finished genomes is exacerbated by sequence repeats and the inability of short reads to capture sufficient genomic information to resolve those problematic regions. In this regard, established and emerging long read technologies show great promise, but their current associated higher error rates typically require computational base correction and/or additional bioinformatics pre-processing before they can be of value.

**Results:**

We present LINKS, the Long Interval Nucleotide *K-*mer Scaffolder algorithm, a method that makes use of the sequence properties of nanopore sequence data and other error-containing sequence data, to scaffold high-quality genome assemblies, without the need for read alignment or base correction. Here, we show how the contiguity of an ABySS *Escherichia coli* K-12 genome assembly can be increased greater than five-fold by the use of beta-released Oxford Nanopore Technologies Ltd. long reads and how LINKS leverages long-range information in *Saccharomyces cerevisiae* W303 nanopore reads to yield assemblies whose resulting contiguity and correctness are on par with or better than that of competing applications. We also present the re-scaffolding of the colossal white spruce (*Picea glauca*) draft assembly (PG29, 20 Gbp) and demonstrate how LINKS scales to larger genomes.

**Conclusions:**

This study highlights the present utility of nanopore reads for genome scaffolding in spite of their current limitations, which are expected to diminish as the nanopore sequencing technology advances. We expect LINKS to have broad utility in harnessing the potential of long reads in connecting high-quality sequences of small and large genome assembly drafts.

**Electronic supplementary material:**

The online version of this article (doi:10.1186/s13742-015-0076-3) contains supplementary material, which is available to authorized users.

## Background

Long-read sequencing technology has rapidly matured over the past few years, and the benefit of long reads for genome assembly is indisputable [1]. Recently, groups have shown that *de novo* assembly of error-rich long reads into complete bacterial genomes is possible [2–4]. Portable long read sequencing technology is at our doorstep, thanks to leaps in microfluidics, electronics and nanopore technologies [5]. Expected to be a strong contender in the kilobase-long read domain, Oxford Nanopore Technologies Ltd (ONT, Oxford, UK) offers a miniature molecule “sensor” that is currently in a limited early access beta-testing phase through the MinION™ Access Programme (MAP). At present, raw uncorrected sequence reads generated by the instrument have limited utility for *de novo* assembly of genomes, which is mostly due to their associated high base errors and indels rates [6]. Recently, Quick and colleagues [6] publicly released ONT *E. coli* long reads as part of the MAP. Although their assessment identified some of the shortcomings of the current technology, it also highlighted its great potential, including a low-cost throughput and kilobase-long reads.

As with any sequencing platform, the ONT data have a unique pattern of correct base calls, mismatches and insertions/deletions (indels). The publicly available datasets utilize the R7 and R7.3 chemistry of the vendor. We observed that under both chemistries, the statistical properties of mismatches and indels follow common profiles, which can be described by mixture models. When we fit the R7 and R7.3 chemistry datasets to these distributions, we observe that they differ in their parameters, but that the structures of the mixture models hold. This is encouraging, as it indicates that the fundamental principles of these distributions can be fixed, and that the datasets can be described by parametric statistical models. It also supports our observation that accurate base calls come in bursts – a property we use in the proposed LINKS algorithm.

**Fig. 1:**
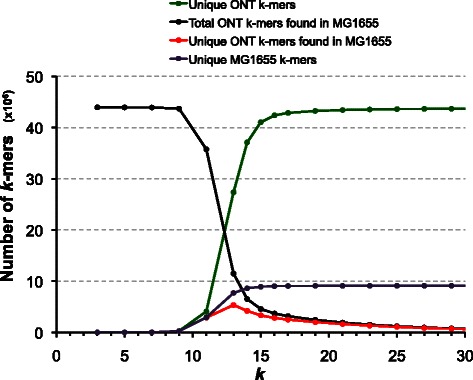
Full 2D ONT long read *k-mer* uniqueness in the *E. coli* K-12 genome reference. *k-*mers were extracted from both the Full 2D R7 ONT data [6] and the *E. coli* K-12 substr. MG1655 (accession U00096.2) reference genome sequence. A Bloom filter [35] was built from the latter and *k*-mers extracted from the former files used to query the filter for matching sequences. *k* = 15 gives the best compromise of specificity, yield and uniqueness with the data set at hand

**Fig. 2:**
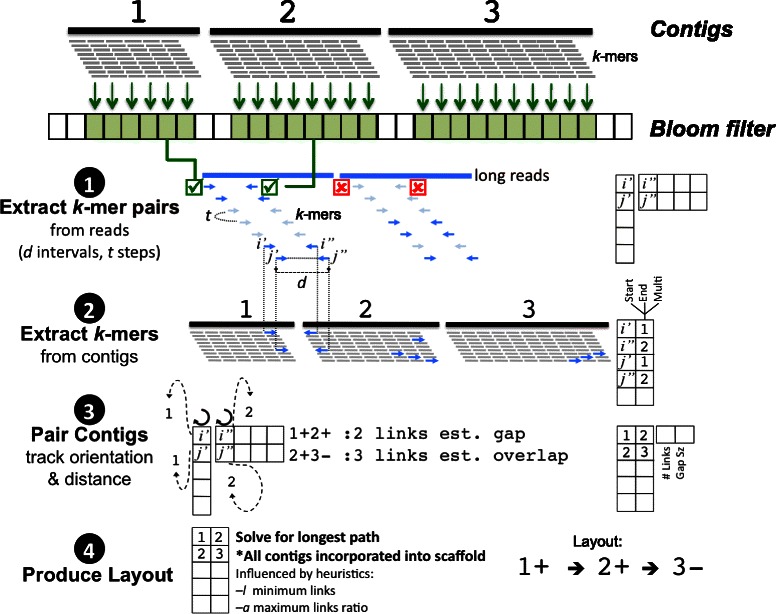
LINKS algorithm. Contigs (three thick black rectangles) are, optionally, shredded into *k-*mers and those *k-*mers used to construct a Bloom filter (green arrows). Long reads (blue rectangles) are processed and *k*-mer pairs *i*’ and *i*” extracted at an interval corresponding to the input distance (−*d*), and window step (−*t*), but stored in memory (step 1, matrix on the right) only if both *k-*mers of a pair (dark blue arrows, green checkmarks) are found in the Bloom filter (step 1). *k-*mers that are not in the Bloom filter are represented by light blue arrows (red checkmark). Contigs are shredded into *k*-mers once more (step 2) using the same *k* value, but stored in memory (step 2, matrix on the right) only when its pair, identified in step 1, exists in memory. In step 3, contigs are paired when *k*-mers are not observed in the same sequence. Iterating through the data structure from step 1 (circular arrows) and verifying placement (Start, End) and multiplicity (Multi) from the data structure in step 2 provides contig linkages (dotted arrows), which are stored into memory (step 3, matrix on the right). In step 4, the scaffold layout is produced by incorporating all contigs into a scaffold, verifying neighbours and merging only when user-defined parameters support it (> = *l* minimum number of links and < = *a* maximum link ratio between alternate-to-primary linkage). In the final layout, the positive symbols following the contig numbers indicate that the contig orientation between the contigs is consistent and unchanged whereas the negative symbol indicates contig 3 on the reverse strand relative to contigs 1 and 2. In this example, contigs 1, 2 and 3 are merged into a single scaffold, with average gap/overlap sizes between contigs calculated using the distance (*d*) between the *k*-mers and their position (*p*) in the respective contigs of length *L* such that the gap (positive value) or overlap (negative value) length = *d* – ((*L*_1_-*p*_1_) + (*p*_2_ + *k*))

For example, we assessed the properties of the high quality R7 chemistry data (termed Full 2D) in the released ONT *E. coli* dataset [6], which comprises reads derived from template and complementary strands. We observed these reads to have an average sequence identity of 77.1 +/− 10.6 % (11 Mbp in 1714 reads with sequence identity of 50 % or more to *E. coli K-*12 MG1655). Despite this arguably low overall quality, there are still frequent continuous stretches of correct *k* bases in the reads when compared to the finished genome. These stretches are long enough to confer specificity, but short enough to be error free (*k* = 15, Fig. 1). We have exploited this property of the sub-5X data (see Additional file 1: Figure S1) to develop LINKS, which extracts paired *k-*mers from the ONT reads, and uses them to link contig pairs (Fig. 2). One advantage and delineating characteristic of the proposed implementation is its ability to iteratively refine assemblies by exploring large numbers of *k-*mer pair combinations for linking contigs, with the potential to process much more linking information than is otherwise possible with sequence-alignment based scaffolding. Here, we show the performance of LINKS for re-scaffolding baseline Illumina *E. coli*, *Salmonella* Typhi and *S. cerevisiae* assemblies using three recently published Oxford Nanopore DNA sequence datasets [6–8]. We also demonstrate the broad applicability and scalability of our tool by re-scaffolding both the *Arabidopsis thaliana* genome with raw and error-corrected Pacific Biosciences of California, Inc. long reads [9, 10] and the massive (20-Gbp) *P. glauca* genome (PG29) using that of another white spruce genotype (WS77111) [11, 12].

## Data description

The datasets supporting the results of this article are available in the GigaDB repository [13] and the European Nucleotide Archive (ENA) under accession number ERP007108 [14] for *E. coli* K-12, at the Figshare repository [15] and ENA accession ERR668747 for *S.* Typhi [16] and at a laboratory public web space for *S. cerevisiae* [17]. The *A. thaliana* assemblies and Pacific Biosciences (PacBio) reads are available online [9, 10, 18]. All LINKS assemblies of small genomes (≤12Mbp) presented herein can be reproduced exactly by downloading LINKS and executing the “runall.sh” script from the package/test repository. For the *A. thaliana* and *P. glauca* LINKS assemblies, we provide bash shell scripts in the package distribution. We also provide each final LINKS assembly ≤12Mbp in the GigaDB repository [19].

## Analyses

A mixture model describes a population of observations assuming that the constituent sub-populations have distinct statistical properties. In this case, the data support the assumption that stretches of mismatched base calls have components that are distributed according to Poisson distributions. We postulate this to be related to the base-calling algorithm, which uses a hidden Markov model. The indels on the other hand have a component with Weibull distribution, which, like Poisson distribution, is commonly used to describe time to “failure” in complex systems. In this case failure corresponds to (i) associating a current level read with a wrong base call (mismatch), (ii) mistaking a fluctuation in the current level to a new base transition through the nanopore (insertion), or (iii) failure to read a change in the measured current (deletion). All error modes have a second component that can be modeled by a geometric distribution, describing by-chance matches between a called base and the base at the corresponding position in the sample DNA. In principle, this is a Bernoulli process, where each trial (test of match/no-match) is independent and identically distributed. Thus these base-calling errors can be statistically described as follows:

$$ \mathrm{Mismatch}:\kern1em {\mathrm{P}}_{\mathrm{m}} \sim {\mathrm{a}}_{\mathrm{m}}\;\mathrm{Poisson}\left({\uplambda}_{\mathrm{m}}\right)+\left(1\hbox{-} {\mathrm{a}}_{\mathrm{m}}\right)\ \mathrm{Geometric}\left({\mathrm{p}}_{\mathrm{m}}\right) $$

$$ \mathrm{Insertion}:\kern1em {\mathrm{P}}_{\mathrm{i}}\sim {\mathrm{a}}_{\mathrm{i}}\;\mathrm{Weibull}\left({\uplambda}_{\mathrm{i}},\ {\upkappa}_{\mathrm{i}}\right)+\left(1\hbox{-} {\mathrm{a}}_{\mathrm{i}}\right)\ \mathrm{Geometric}\left({\mathrm{p}}_{\mathrm{i}}\right) $$

$$ \mathrm{Deletion}:\kern1.25em {\mathrm{P}}_{\mathrm{d}}\sim {\mathrm{a}}_{\mathrm{d}}\;\mathrm{Weibull}\left({\uplambda}_{\mathrm{d}},\ {\upkappa}_{\mathrm{d}}\right) + \left(1\hbox{-} {\mathrm{a}}_{\mathrm{d}}\right)\ \mathrm{Geometric}\left({\mathrm{p}}_{\mathrm{d}}\right) $$

Here a_x_ ∈ (0, 1) are mixture parameters, λ_m_ represents the expected value of the Poisson distribution, p_x_ are the Bernoulli trial probabilities for the geometric distributions, and l_x_ and κ_x_, respectively, are the scale and shape parameters of the Weibull distributions (see Additional file 1: Table S1). Our substitution profile is generated based on LAST [20] alignments and it is consistent with recent reports looking at error models of ONT reads derived from the *E. coli* phage M13 [21]. Although the substitution rate is much higher for *E. coli* K-12 2D reads, the general trend in transition probability is the same as for the M13 phage. For instance, A and T are more stable than C and G and bases are generally more likely to be substituted by C and G during sequencing. We highlight that modeling errors will be influenced by parameterization and choice of alignment software. However, the underlying error models derived here support visual inspections of ONT-to-reference pair-wise nucleotide sequence alignments, that is, stretches of accurate bases interspersed with bursts of indels and/or mismatched bases. These correct-matching base stretches are of length 13–15 bp on average, long enough to confer specificity (Fig. 1). Pairing such *k* stretches at given distance intervals and comprehensively exploring the paired *k-*mer space effectively compensates for the high-error rate currently observed in these data, is the basis of our method and a strategy transferable to higher quality DNA reads or sequences, as we show below.

To demonstrate the performance of LINKS, we used two publicly available ONT datasets [6] (R7 and R7.3 chemistry) for scaffolding *E. coli* contigs, and scaffolds derived from high-depth Illumina MiSeq (San Diego, CA) 300 bp paired end reads that are assembled with ABySS [22]. These assemblies were already very contiguous (scaffold NG50 [23] = 204 kbp) and of good quality, as assessed by QUAST [24] (Table 1). First, we scaffolded ABySS contigs (Table 1A) with only the ONT Full 2D data (*k* = 15, *d* = 4000), as a benchmark of the method, yielding an improved assembly (Table 1C, Additional file 1: Figure S2) that rivaled the ABySS scaffolder on Illumina data in assembly quality (Table 1B). The runs were fast (~1 min), and required moderate resources (~2.1 GB RAM). Next, we re-scaffolded *E. coli* ABySS scaffolds using the same parameters (Table 1D, Additional file 1: Figure S3). LINKS generated 4,891,397 *k-*mer pairs, but only 68,363 (1.4 %) of those were found both to be unique and to pair unambiguously in the scaffold (Table 1B) assembly. Encouragingly, 54,953 (80.3 %) satisfied the pairing (*k-*mer pairs facing inwards) and distance (4,000 +/− 400 bp) logic imposed on the *k-*mers, with fewer than 700 not satisfying the expected pairing or distance logic. The larger portion of conflicts originated from contig pairs, with 12,033 having a calculated distance beyond the maximum allowed distance (such as a calculated distance of 4,400 bp, indicative of overlaps 400 bp or greater between contigs). Running SSPACE-LongRead [25] (Table 1E) gave comparable results to that of LINKS (Table 1D), producing a marginally less contiguous assembly, but using less memory by a factor of 3.

**Table 1:** QUAST analysis of a baseline *E. coli* assembly and re-scaffolded assemblies using Oxford Nanopore 2D (R7 chem.) or raw (R7.3 chem.) reads

Stats based on sequences ≥ 500 bp	A. ABySS contigs	B. ABySS scaffolds	C. LINKS k15d4000	D. LINKS k15d4000	E. SSPACE-LR g200	F. LINKS x30 k15d500–16kbp	G. LINKS x30 k15d500–16kbp	H. LINKS x30 k15d500–16kbp
Input data	Illumina MiSeq	A. +MiSeq	A. +ONT Full 2D R7	B. + ONT Full 2D R7	B. +ONT Full 2D R7	B. +ONT Full 2D R7	B. +ONT All 2D R7	B. +raw ONT R7.3
Read input fold coverage	241.2x	241.2x	4.7x	4.7x	4.7x	4.7x	34.2x	67.0x
Time (h:mm:ss)	-	-	0:01:35	0:01:32	0:01:09	0:17:43	1:50:25	3:04:11
Memory (GB)	-	-	2.1	2.1	0.7	4.3	27.2	46.7
Total sequences	67	61	49	48	43	27	16	26
Largest (bp)	358,719	406,793	633,204	633,147	628,411	1,057,556	1,286,148	1,286,419
NG50 length (bp)	179,720	206,356	270,992	293,925	226,696	633,147	1,197,321	645,796
Misassemblies	5	5	5	5	8	11	20	9
Genes + parts	4,442 + 63	4,443 + 62	4,443 + 62	4,443 + 62	4,448 + 57	4,443 + 62	4,440 + 62	4,443 + 62
Max.alignment (bp)	358,223	405,659	486,572	486,527	405,659	760,934	760,934	759,131
Number of N’s per 100 kbp	109.74	113.61	189.32	192.88	388.10	325.95	566.97	401.61
NGA50 (bp)	177,531	179,569	228,879	228,879	226,324	299,206	299,206	486,527
NA50 (bp)	146,850	177,531	226,324	226,324	215,056	293,772	294,667	344,280

**Fig. 3:**
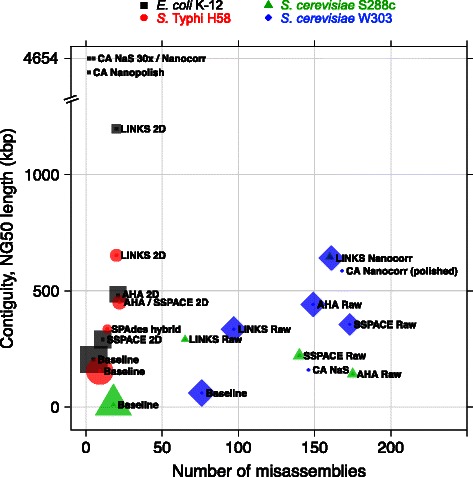
Scaffolding high-quality short read assemblies with Oxford Nanopore Technologies long reads. Publicly available ONT long reads for *E. coli* K-12 MG1655, *S.* Typhi and *S. cerevisiae* W303 were recently made available [6–8]. We have used these data to re-scaffold *E. coli* K-12, *S. cerevisiae* S288c and *S. cerevisiae* W303 baseline assemblies of Illumina-only data using LINKS, AHA and SSPACE-LR and assessed the quality of the resulting assemblies by plotting the NG50 length contiguity and number of misassemblies reported by QUAST [24] (black square, green triangle, blue diamond). Also, we have re-scaffolded a baseline *S.* Typhi Illumina assembly iteratively (11x) with LINKS using R7 2D ONT data and compared it to the SPAdes hybrid Illumina + ONT assembly reported [7] (red circles). We compare the results to Celera Assemblies (CA) of Illumina-corrected ONT reads (NaS and Nanocorr polished) and ONT-corrected ONT reads (Nanocorrect/Nanopolish) [4, 8, 26]. The re-scaffolding software ran on either all R7 chemistry 2D (2D), raw or Nanocorr-corrected reads, as indicated. For *S.* Typhi, the AHA and SSPACE-LR re-scaffolded assemblies were comparable and their corresponding data points overlapped (refer to Table 2). Data point size is normalized on the number of resulting scaffolds in each experiment. Smaller points indicate a better outcome (less scaffolds)

LINKS can identify further merge opportunities by comprehensively extracting paired *k-*mers at various distance intervals. Accordingly, we ran our tool iteratively 30 times and at each instance increased the distance between *k-*mer pairs from 500 to 16,000 bp, thus gradually improving the long-range contiguity of the assembly (Table 1F, Additional file 1: Figure S4). The iterative runs had a relatively longer runtime (~18 min.) to generate a final assembly with less than half the original number of sequences, and a scaffold NG50 length exceeding 600 kbp. With the same run parameters, using all available 2D ONT data, LINKS required 27.2 GB RAM and 1h50m to complete (Table 1G), and yielded 12 scaffolds >1 kbp that are co-linear with the *E. coli* genome (Additional file 1: Figure S5). Using raw R7.3 ONT *E. coli* reads for scaffolding gave the best compromise between errors and contiguity (Table 1H, Additional file 1: Figure S6, Table 2). Recent reports present ONT read-error correction technologies that are applied prior to assembly with the Celera Assembler, using either Illumina reads or the ONT reads themselves [4, 8, 26, 27]. Corresponding *E. coli* K-12 assemblies are near perfect (Fig. 3), but require sufficient read depth, potentially long compute time (in our hands Nanocorrect/Nanopolish ran for ~4.5 days on a 12-core computer) and it is unclear at the moment how these error-correction methods will scale to larger genomes when the ONT sequencing allows it. In this study, we have concentrated on using the results of a single run of ONT *E. coli* reads, which provided ~34-fold and <5-fold coverage of the genome with 2D and high-quality (Full) 2D reads, respectively (Additional file 1: Figure S1). This is in contrast with the study reporting on Nanocorrect/Nanopolish [26] that utilized data from four ONT R7.3 runs, providing ~29-fold theoretical coverage (2D reads) of the 4.6 Mbp *E. coli* K-12 genome.

**Table 2:** QUAST analysis of baseline and re-scaffolded *E. coli* K-12, *S.* Typhi H58, *S. cerevisiae* S288c and W303 assemblies using Oxford Nanopore Technologies reads

Genome	Method	Data/Chemistry/ Fold coverage	Number of contigs (> = 500 bp)	NG50 length (bp)	NA50 length (bp)	Number of genes	Number of N’s per 100 kbp	Number of mis-assemblies	Mis-assemblies type relocations/Trans-locations/Inversions
*E. coli* K-12	*Baseline*	*Illumina*	*61*	*206,356*	*177,531*	*4,443*	113.61	*5*	*5/0/0*
	AHA	F2D/R7/4.7x	46	226,696	179,569	4,441	390.31	12	12/0/0
		2D/R7/34.2x	34	480,126	266,663	4,441	719.89	21	21/0/0
		Raw/R7.3/67.0x	30	762,313	344,280	4,443	523.61	9	9/0/0
	LINKS	F2D/R7/4.7x	27	633,147	293,772	4,443	325.95	11	11/0/0
		2D/R7/34.2x	16	1,197,321	294,667	4,440	566.97	20	20/0/0
		Raw/R7.3/67.0x	27	645,797	344,280	4,443	401.61	9	9/0/0
	CA-Nanocorr	Raw/R7/145x	**1**	**4,654,420**	2,768,544	**4,515**	**0.00**	5	4/0/1
	CA-Nanopolish	2D/R7.3/29x	**1**	4,593,653	2,739,950	**4,515**	**0.00**	**2**	**2/0/0**
	CA-NaS	2D/R7/30x	**1**	4,654,321	**3,507,873**	**4,515**	**0.00**	**2**	**2/0/0**
	SSPACE-LR	F2D/R7/4.7x	43	226,696	215,056	4,448	388.10	8	8/0/0
		2D/R7/34.2x	37	300,940	216,050	4,446	682.61	11	11/0/0
		Raw/R7.3/67.0x	39	238,960	226,324	4,448	539.84	8	8/0/0
*S.* Typhi H58	*Baseline*	*Illumina*	*86*	*153,674*	*114,216*	*4,305*	*0.00*	*9*	*8/0/1*
	AHA	2D/R7/4.7x	38	472,758	267,543	4,308	213.33	22	20/0/2
	LINKS		**22**	**652,465**	**332,188**	4,313	434.83	21	20/0/**1**
	SPAdes		34	319,375	282,035	**4,344**	**0.00**	**14**	**12**/0/2
	SSPACE-LR		29	473,660	280,966	4,331	236.65	21	20/0/**1**
*S. cerevisiae* S288c	*Baseline*	*Illumina*	*1,673*	*10,038*	*10,671*	*5,117*	*86.66*	*18*	*8/10/0*
	AHA	Raw/R7/119.9x	363	141,253	78,176	5,325	4,996.13	173	127/46/0
	LINKS		**189**	288,075	**173,906**	5,269	3,989.86	**66**	**23/43/0**
	LINKS-Nanocorr^a^	Nanocorr/40.3x	224	**645,967**	150,299	5,282	15,469.63	160	72/88/0
	SSPACE-LR	Raw/R7/119.9x	472	220,727	129,088	**5,363**	10,646.35	140	32/106/2
*S. cerevisiae* W303	*Baseline*	*Illumina*	*4,021*	*59,927*	*49,258*	*6,018*	*3,989.86*	*76*	*20/56/0*
	AHA	Raw/R7/119.9x	3,706	440,962	168,384	6,040	1603.59	149	43/106/0
	LINKS		3,782	334,705	176,649	6,030	784.78	**97**	**27/72/0**
	LINKS-Nanocorr^a^	Nanocorr/40.3x	3,671	**640,472**	165,312	6,035	2,171.54	161	41/120/0
	CA-Nanocorr		95	585,890	263,428	6,247	**0.00**	166	76/90/0
	CA-Nanocorr (polished)		95	585,932	**263,474**	**6,250**	**0.00**	168	77/90/1
	CA-Nanocorr (polished)-LINKS^a^	Raw/R7/119.9x	**93**	585,932	**263,474**	**6,250**	24.05	169	80/88/1
	CA-NaS	13x of >10 kb reads	121	159,881	109,441	6,016	**0.00**	146	76/70/0
	SSPACE-LR	Raw/R7/119.9x	3,249	354,977	126,601	6,044	10,953.41	173	41/132/0

^a^LINKS-Nanocorr are derived from re-scaffolding baseline Illumina assemblies with Nanocorr-corrected ONT reads whereas CA-Nanocorr (polished)-LINKS is derived from re-scaffolding the CA-Nanocorr (polished) assembly [8, 17] with raw ONT W303 reads

^b^Best genome assembly metrics are highlighted in Bold

Similarly to *E. coli*, we ran LINKS iteratively to re-scaffold a baseline Illumina assembly of *Salmonella enterica* serovar Typhi (*S.* Typhi) haplotype H58 with 2D ONT reads, and compared the results to those provided by the authors [7]. The study authors report a marked improvement in assembly contiguity when assembling concurrently Illumina and ONT reads (34 contigs, 319 kbp N50 length), when compared to a baseline assembly of Illumina-only data (86 contigs, 154 kbp N50 length), consistent with our assessment of their data. The final LINKS assembly on this dataset, took 21 min. and used 8.2 GB RAM to yield 22 contigs with an N50 length of 652 kbp, approximately double the contiguity previously reported [7]. Testing LINKS on the larger *S. cerevisiae* W303 ONT dataset [8], we obtained an assembly that compares with the Celera Assembly of Illumina-corrected ONT reads (Nanocorr) in contiguity, but with 40 % less errors than the Pilon-polished [28] Celera assembly (Fig. 3, Table 2). It is worth noting, however, that LINKS is a scaffolder and as such, merged contigs are ordered and oriented within scaffolds, separated by gaps/overlaps and that its resulting W303 assembly, much like that of other scaffolders, comprises over 3700 scaffolds, versus only 95 and 121 for the resulting Celera Assembly (CA) assembly of Nanocorr and Nanopore Synthetic-long (NaS) reads, respectively. When scaffolding the W303 baseline Illumina assemblies with Nanocorr-corrected ONT reads, we notice that the error count of the resulting LINKS assembly is marginally less than the polished Celera assembly, albeit higher (2.6-fold) than a LINKS assembly re-scaffolded with uncorrected reads, which indicates that Nanocorr-corrected reads may introduce errors that are propagated during assembly and/or scaffolding of the yeast data (Fig. 3). The quality of the resulting LINKS assembly depends on a few factors, including the quality of the input assembly and the stringency of the imposed linkage, which was fairly relaxed in this study (e.g., minimum 5 links). We assessed misassembly types, using the breadth of the ONT data presented and observed that LINKS outperforms all methods on the larger yeast data, including Nanocorr. Even though it introduces more relocation errors in *S.* Typhi (errors caused by gap/overlap size estimates over/under 1kbp), compared to SPAdes (St. Petersburg genome assembler), it never introduces inversions (Table 2).

While LINKS uses large amounts of memory with increased target genome sizes, this can be mitigated by the Bloom filter implementation (LINKS v1.5), which decreases the RAM usage 3-fold compared to earlier versions. With all versions of LINKS, a smaller memory footprint is achieved by increasing the sliding window step (−*t*) and augmenting the distance between *k*-mer pairs (−*d*), which in turn decreases the *k-*mer pair space. Because LINKS is a scaffolder, it may be used downstream of other assembly methodologies, as exemplified on the *S. cerevisiae* W303 data, where two additional merges from the polished CA + Nanocorr assembly were made using raw W303 ONT reads (Table 2).

We demonstrate the broad applicability of LINKS, by re-scaffolding a high-quality draft of the 120-Mbp *A. thaliana* Ler-1 genome [9] with either raw or corrected [10, 18] long sequence reads from PacBio. We find that *k* = 21 worked best with this data, and that lower *k* values (*k* = 13 and 15 explored), did not merge any scaffolds due to increased conflicts in contig pairs (not shown). Iterative scaffolding (4 iterations) using a low sliding window for 3 out of the 4 iterations (−*t* 5) completed in 1h52m and required 84 GB using the 118-fold raw PacBio data set and 3h05m and 151 GB RAM with the lower depth (28-fold) ECTools-corrected PacBio reads. The increased resource requirements are not surprising given that error correction yields 135.9 M *k*-mer pairs from 288,217 reads, which is more than 3 orders of magnitude compared to the 1.33 M extracted from the 3.45 M raw PacBio reads (not shown). We find that the resulting LINKS assemblies are very contiguous, especially when the PacBio reads are corrected (NG50 > 2.5 Mbp), and highlights 1) the utility of LINKS for retrospective scaffolding of draft genomes with new long read sequencing data and that 2) LINKS scaffolding can be complimentary to read correction methodologies (Additional file 1: Figure S7). When compared to other assemblies of PacBio-only data, we find that the final LINKS assemblies of high-quality Illumina draft assemblies tend to harbor fewer errors, as also demonstrated on the yeast data (Fig. 3, Table 2, Additional file 1: Figure S8 and Table S2). Four LINKS iterations performed on a baseline Illumina assembly with raw PacBio reads representing 118-fold coverage increased the NG50 length over 8-fold from 59 to 492 kbp. The use of ECTools-corrected PacBio reads [10] further increased the contiguity, as measured by the NG50 length (765.4 kbp), but also yielded 284 additional misassemblies compared to the 4th and final LINKS iteration that used raw PacBio reads. The Illumina Allpaths-LG assembly [9, 29] was already very contiguous at 310.7 kbp NG50 length, but re-scaffolding with the same raw and ECTools-corrected PacBio data increased the NG50 length to 1.45 and 2.65 Mbp, respectively. This is in contrast to the ECTools and PacBioToCA assemblies (NG50 = 487.2 and 370.7 kbp, in this order) still three times lower in assembly contiguity compared to The Hierarchical Genome Assembly Process (HGAP) (NG50 = 8.429 Mbp). Evidently, because LINKS re-scaffolded assemblies are derived from fragmented Illumina draft assemblies, they contain ambiguous bases (Ns) when compared to their PacBio-only counterparts. However, the contiguity metric normalized on genome size and that accounts for assembly error, the NGA50 length, is similar (87.5 vs. 78.0 kbp) between the highly contiguous HGAP PacBio-only assembly and the Allpaths-LG Illumina assembly re-scaffolded with LINKS using ECTools-corrected reads, which suggests that LINKS offers a good compromise between contiguity and errors, in a lightweight and easy to use software package (Additional file 1: Figure S8 and Table S2).

**Fig. 4:**
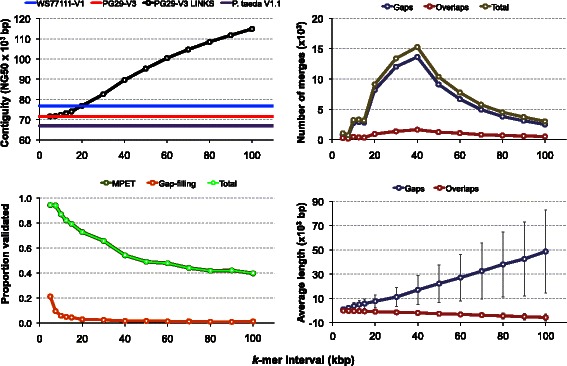
LINKS re-scaffolding of the white spruce (*P. glauca*, PG29 cultivar) genome with *k*-mer pairs derived from the white spruce WS77111 genotype draft assembly. Iterative LINKS scaffolding rounds (v1.1 with fourteen iterations, k = 26, t = 200 to 50, l = 5, a = 0.3, d = 5 kbp to 100kbp, interval shown on x axis, solid black line) were performed on the PG29 V3 ABySS assembly sequence scaffolds (Genbank: GCA_000411955.3, 4.2 M scaffolds ≥ 500 bp, bottom left panel, red line) using sequence data from the WS77111 V1 draft assembly (Genbank: PRJNA242552, 4.3 M scaffolds ≥ 500 bp, top left panel, blue line), making 84,529 total merges (top right panel) and increasing the PG29 assembly contiguity 1.5-fold to reach an NG50 length [23] of 114,888 bp (4.1 M scaffolds ≥ 500 bp; top left panel). We have validated the final LINKS assembly of spruce with scalable gap-filling software Sealer [34] and MPET reads from 4, 8 and 12kbp libraries (bottom left panel). Validation with the latter and its diminished return with large *k*-mer intervals tracks with the increase in gap lengths (bottom right panel). We note that LINKS re-scaffolding of the white spruce assembly was done to demonstrate scalability

We demonstrate the scalability of our algorithm by iteratively re-scaffolding the colossal 20-Gbp white spruce genome (PG29) draft [11, 12] 14 times, using the assembly of another white spruce genotype (WS77111) as long-read input. We had previously profiled structural variations between the two drafts genomes and found little sequence divergence between the two genotypes [12], providing the impetus of the re-scaffolding work presented here. This iterative LINKS run had a peak memory of ~ 132 GB RAM, producing a conifer genome assembly whose NG50 contiguity is in excess of 110 kbp. We validated the white spruce LINKS assemblies at each *k*-mer interval using gap-filling software and sequence alignment of long-range (4, 8 and 12 kbp) mate pair (MPET, a.k.a. jumping) sequence data, and observe a decreased validation rate that is consistent with increased gap length over large paired *k*-mer distances (Fig. 4). We validated the LINKS assemblies at each *k*-mer interval using gap-filling software and sequence alignment of long-range (4, 8 and 12 kbp) MPET sequence data, and observe a decreased validation rate that is consistent with increased gap length over large paired *k*-mer distances (Fig. 4). Altogether, 49,532 out of the 84,529 total LINKS merges (58.6 %) were validated by MPET reads (Additional file 2). We do not expect complete validation of merges due to the limiting fragment length of the MPET library (max. 12 kbp) and average gap sizes introduced at large intervals (48.6 ± 34.2 S.D. kbp, at the 14th iteration). This shows that not only LINKS scales to one of the largest genome sequenced (20 Gbp), it does so correctly. We launched SSPACE-LR on the same data, and from the file size (8 GB) written to disk after 3 weeks, we estimated the process to be 1/3 complete at that time. This process executed despite an error message stating that read files larger than 4 GB are not supported by SSPACE-LR.

## Discussion

In recent months, there have been advances in correcting ONT reads [4, 8, 26], which makes the resulting, corrected, long reads suitable to assembly with established overlap layout consensus assembly software [27]. It is important to note that both the Nanocorr and Nanocorrect/Nanopolish ONT long read correction methods are not assembly methodologies *per se*, but base error correction utilities and as such, the resulting error-corrected reads they produce can be readily used by LINKS to contiguate pre-existing genome assemblies. Likewise, LINKS is a genome scaffolder, not a sequence assembler, and does not attempt to correct assembly bases or fill Ns that result from its merges. Like other scaffolding algorithm before it, it orders and orient contigs into larger scaffolds that could be used to characterize genomic loci of interest. The novelty of the algorithm lays in its scalability and usage of paired *k*-mers from varied long sequence sources (Oxford Nanopore Technologies, Pacific Biosciences, draft sequences), without the need to correct read bases first.

As larger genomes are sequenced with ONT and PacBio, larger *k*-values will be needed to disambiguate linkages that would otherwise likely happen by chance at the low value of *k* (*k* = 15) used herein. However, using larger *k*-mers may not be possible when using the current R7 and R7.3 chemistries of ONT, given the error models we derived and present here. However rapid improvements in chemistries, base calling and error-correction algorithms already indicate that this is unlikely to be a problem for the broad applicability of LINKS to larger genomes, using a diverse long-read source. This is exemplified here in our use of raw and error-corrected PacBio long reads (*k* = 21) to re-scaffold the 120 Mbp *A. thaliana* genome and the use of a genotype assembly draft of white spruce (*k* = 26) to re-scaffold the 20-Gbp *P. glauca* genome.

LINKS is a scalable, alignment-free scaffolder, which extracts spaced *k-*mers from reads as its pairing information source to order and orient sequence contigs into scaffolds. It takes input reads from a variety of sources, including ONT and PacBio sequences, but as demonstrated, it can also work with other long sequences to contiguate assemblies. It offers a general framework that could apply to scaffolding very large genomes, such as that of white spruce using another assembly draft or reference in lieu of long reads. This study also highlights the present utility of ONT reads for genome scaffolding in spite of their current limitations, which are expected to diminish as nanopore sequencing technology advances. LINKS is available for public use [30].

## Methods

### Sequence data, assembly, and scaffolding

*E. coli K-*12 substrain MG1655 Illumina MiSeq v3 TruSeq Nano read data (paired end 301 bp, fragment length 550 bp) was downloaded from BaseSpace®, and randomly sub-sampled to ~250-fold coverage. Overlapping read pairs were merged with ABySS-mergepairs (−q 15) and resulting ca. 550 bp pseudoreads were assembled with ABySS v1.5.2 [22] (*k =* 480 *l =* 40 *s =* 1000) yielding 67 and 61 contigs and scaffolds ≥ 500 bp, respectively. Contigs and scaffolds (Table 1A and B, Additional file 1: Figure S2) were scaffolded with LINKS v1.5 (*k =* 15, *d =* 4000, default parameters) using the *E. coli K-*12 substr. MG1655 R7 Full 2D ONT data from Quick and colleagues [6] (R7 chemistry ONI/NONI ENA:ERX708228), and results are shown in Table 1C, Additional file 1: Figure S3 and Table 1D, Additional file 1: Figure S4, in that order. SSPACE-LongRead [25] v1.1 (abbreviated SSPACE-LR. Options: g = 200, with defaults parameters) ran on the Table 1B assembly (Table 1E). ABySS scaffolds were also re-scaffolded iteratively with LINKS (v1.5, *k =* 15, *d =* 500 to 16000, 30 iterations) using the Full 2D ONT reads (Table 1F) and, in separate experiment, all available 2D reads (Table 1G, Additional file 1: Figure S5) and all available R7.3 chemistry raw uncorrected FASTA reads derived from poretools [31] conversion (ENA:ERX593921;Table 1H, Additional file 1: Figure S6). A baseline *S.* Typhi H58 Illumina assembly [7] (Genbank:GCA_000944835.1) was re-scaffolded with LINKS (v1.5, *k =* 15, *d =* 500 to 4000, *t* = 1, *a* = 0.1, 11 iterations) using 2D ONT reads (ENA:ERR668747). A Baseline *S. cerevisiae* W303 Illumina MiSeq assembly [8, 17] and *S. cerevisiae* S288c were respectively re-scaffolded with SSPACE-LongRead (g = 200), A Hybrid Assembler (AHA) [32] and LINKS (v1.5, *k =* 15, *d =* 2-15kbp, 27 or 29 iterations) using 262,463 raw ONT reads (Fig. 3). The baseline *A. thaliana* Ler-1 Allpaths-LG assembly [9] was re-scaffolded with LINKS (v1.5, *t* = 20|5|5|5, *k =* 21, *d = 5*–20kbp, 4 iterations) using 19 SMRTcells of corrected (ECTools [10]) or 93 SMRTcells PacBio raw reads totaling 14.2 GB of data and providing 118-fold coverage of the genome, 38-fold from reads 10 kbp or larger [18] (Additional file 1: Table S2). PacBio assemblies used for comparison were downloaded [18] and assessed with QUAST [24] using the reference *A. thaliana* TAIR10 genome (Genbank:GCA_000001735.1). The 20-Gbp white spruce [11, 12] V3 assembly (Genbank:ALWZ030000000, 4.2 M scaffolds) was re-scaffolded with LINKS 14 times (v1.1, *k =* 26, *t =* 200–50 *d =* 5–100kbp) using the draft white spruce WS77111 V1 genotype assembly (Genbank:JZKD010000000, 4.1 M sequences) (Fig. 4). The white spruce MPET libraries used for validation are presented in [11] and available from the dnanexus repository [33]. Validation of merges by automated gap closure was done with the scalable gap-filling software Sealer, using the same parameters described in a recent publication [34], performed on the final 14th re-scaffolded LINKS assembly (Additional file 2). All benchmarking was done on a computer with Intel(R) Xeon(R) CPU E5-2699 v3 at 2.30GHz, 72 CPUs with 264 GB RAM.

### Algorithm

FASTA sequences to scaffold are supplied as input (−*f*), and are shredded to *k-*mers on both strands, populating a Bloom filter [35] whose number of elements corresponds to a rough approximation of the number of *k*-mers in the draft genome based on file size. The size of the filter can be adjusted by controlling its false positive rate (−*p*). Building a Bloom filter is optional (−*x*), but strongly recommended as it decreases the memory usage and run time when tested on smaller genomes (<20 Mb). For large genomes (≥1 Gb), we recommend pre-building the Bloom filter with the supplied utility (./tools/writeBloom.pl in the distribution). ONT reads are supplied as input (−*s* option, file-of-filenames listing FASTA/FASTQ formatted files) and *k-*mer pairs are extracted using user-defined *k-*mer length (−*k*) and distance between the 5’-end of each pairs (−*d*) over a sliding window (−*t*). When both *k-*mers are found in the Bloom filter, unique *k-*mer pairs at set distance are hashed, tracking the contig or scaffold of origin, *k-*mer positions and frequencies of observation. LINKS has two main stages: contig pairing, and scaffold layout. Cycling through *k-*mer pairs, *k-*mers that are uniquely placed on contigs are identified. Putative contig pairs are formed if *k-*mer pairs are on different contigs. Contig pairs are only considered if the calculated distances between them satisfy the mean distance provided (−*d*), while allowing for a deviation (−*e*). Contig pairs having a valid gap or overlap are allowed to proceed to the scaffolding stage. Contigs in pairs may be ambiguous: a given contig may link to multiple contigs. To mitigate, the number of spanning *k-*mer pairs (links) between any given contig pair is recorded, along with a mean distance estimate. Once pairing between contigs is complete, the scaffolds are built using contigs in turn until all have been incorporated into a scaffold. Scaffolding is controlled by merging sequences only when a minimum number of links (−*l*) join two contig pairs, and when links are dominant compared to that of another possible pairing (−*a*). The predecessor of LINKS is the unpublished scaffolding engine in the widely used SSAKE assembler [36], and foundation of the SSPACE-LongRead scaffolder [25]. A summary of the scaffold layout is provided (.scaffold) as a text file, and captures the linking information of successful scaffolds. A FASTA file (.scaffold.fa) is generated using that information, placing N-pads to represent the estimated lengths of gaps, and a single “n” in cases of overlaps between contigs. A log summary of *k-*mer pairing in the assembly is provided (.log) along with a text file describing possible issues in pairing (.pairing_issues), pairing distribution (.pairing_distribution.csv) and compressed Bloom filter (.bloom). The Bloom filter is intended to be re-used (supplied via -*r*) for iterative LINKS runs.

### Statistical modeling

2D ONT reads from a single run (ERX708228) were aligned to reference genome using LAST [20] (v581, options: −a 1 -r1 -b1), consistent with that of other reports [6, 21]. Only the best alignment of each query sequence was chosen, and alignments were clipped from both ends to the start of the first match and the end of the last match positions. Each clipped alignment is composed of match, mismatch, insertion and deletion fragments. The lengths of these fragments were tallied, and mismatch fragment lengths were stored as zero-indexed values, while the indels were stored as one-indexed values to model interarrival times of “failures”. The model fitting was performed using R. All proposed mixture model fits were tested using Kolmogorov–Smirnov tests with a p-value threshold of 0.05.

### Availability and requirements

**Project name:** Long Interval Nucleotide K-mer Scaffolder

**Project home page:**http://www.bcgsc.ca/bioinfo/software/links and https://github.com/warrenlr/LINKS/

**Operating system:** Unix, Mac OS X

**Programming language:** PERL

**Other requirements:** Unix

**License:** GNU General Public License - GPL.

## Availability of supporting data

The datasets supporting the results of this article and snapshots of the code are available in the GigaDB repository [19]. The public data used in this study is summarized in Additional file 1: Table S3 and S4.

## Additional files

Additional file 1: **Additional table and figures.** **Table S1.** Statistical models of base errors in R7 and R7.3 Oxford Nanopore Technologies long reads. **Figure S1.** *E. coli* K-12 substr. MG1655 genome coverage analysis by Full 2D (R7 chemistry) Oxford Nanopore long reads. **Figure S2.** *E. coli* K-12 Illumina baseline assembly and genome co-linearity. **Figure S3.** Full 2D ONT - LINKS scaffolds co-linearity with the MG1655 genome, single *k*-mer pair LINKS run. **Figure S4.** Full 2D ONT-LINKS scaffolds co-linearity with the reference *E. coli* K-12 genome (thirty *k-*mer pair interval iterations). **Figure S5.** LINKS scaffolds using all available R7 2D ONT reads compared to the reference *E. coli* K-12 genome (thirty *k-*mer pair interval iterations). **Figure S6.** LINKS scaffolds using all raw, uncorrected R7.3 ONT reads compared to the reference *E. coli* K-12 genome (thirty *k-*mer pair interval iterations). **Figure S7.** LINKS re-scaffolding of *A. thaliana* Ler-1 genome draft using raw and ECTools-corrected PacBio long reads. **Figure S8.** LINKS assemblies of baseline *A. thaliana* Ler-1 or Ler-0 genome drafts using raw and ECTools-corrected PacBio long reads. **Table S2.** QUAST analysis of LINKS re-scaffolded A. thaliana Illumina-only assemblies compared to public assemblies of Pacific Biosciences data. **Table S3.** Read data used for scaffolding. **Table S4.** Baseline assemblies used for scaffolding. (DOCX 1119 kb) Additional file 2: **Iterative scaffolding of the 20 Gbp white spruce genome and validation of gaps with MPET data.** (XLS 26 kb)

## Abbreviations

CA: Celera Assembler
ENA: European Nucleotide Archive
LINKS: Long Interval Nucleotide K-mer Scaffolder
MAP: MinION™ Access Programme
NaS: Nanopore Synthetic-long reads
ONT: Oxford Nanopore Technologies
PacBio: Pacific Biosciences
RAM: Random Access Memory
