# Supplementary material for: LINKS: Scalable, alignment-free scaffolding of draft genomes with long reads
Source: Gigascience. 2015 Aug 4;4:35. doi: 10.1186/s13742-015-0076-3 (PMC4524009; doi:10.1186/s13742-015-0076-3)
Supplement: Additional file 1: — Additional table and figures. Table S1. Statistical models of base errors in R7 and R7.3 Oxford Nanopore Technologies long reads. Figure S1. E. coli K-12 substr. MG1655 genome coverage analysis by Full 2D (R7 chemistry) Oxford Nanopore long reads. Figure S2. E. coli K-12 Illumina baseline assembly and genome co-linearity. Figure S3. Full 2D ONT - LINKS scaffolds co-linearity with the MG1655 genome, single k-mer pair LINKS run. Figure S4. Full 2D ONT-LINKS scaffolds co-linearity with the reference E. coli K-12 genome (thirty k-mer pair interval iterations). Figure S5. LINKS scaffolds using all available R7 2D ONT reads compared to the reference E. coli K-12 genome (thirty k-mer pair interval iterations). Figure S6. LINKS scaffolds using all raw, uncorrected R7.3 ONT reads compared to the reference E. coli K-12 genome (thirty k-mer pair interval iterations). Figure S7. LINKS re-scaffolding of A. thaliana Ler-1 genome draft using raw and ECTools-corrected PacBio long reads. Figure S8. LINKS assemblies of baseline A. thaliana Ler-1 or Ler-0 genome drafts using raw and ECTools-corrected PacBio long reads. Table S2. QUAST analysis of LINKS re-scaffolded A. thaliana Illumina-only assemblies compared to public assemblies of Pacific Biosciences data. Table S3. Read data used for scaffolding. Table S4. Baseline assemblies used for scaffolding. (DOCX 1119 kb) [file 13742_2015_76_MOESM1_ESM.docx]

**Supplementary material (Additional File 1)**

**Table S1. Statistical models of base errors in R7 and R7.3 Oxford Nanopore Technologies long reads**

|  | Mismatch | | | Insertion | | | | Deletion | | | |
| --- | --- | --- | --- | --- | --- | --- | --- | --- | --- | --- | --- |
|  | *a_m_* | *λ_m_* | *p*_m_ | *a_i_* | *λ_i_* | *κ_i_* | *p_i_* | *a_d_* | *λ_d_* | *κ_d_* | *p_d_* |
| *E. coli* R7 | 0.248 | 0.480 | 0.711 | 0.850 | 0.968 | 1.004 | 0.418 | 0.870 | 1.023 | 0.986 | 0.403 |
| *E. coli* R7.3 | 0.138 | 0.441 | 0.476 | 0.900 | 1.473 | 1.045 | 0.272 | 0.959 | 1.682 | 1.059 | 0.249 |
| *S. cerevisiae* R7 | 0.177 | 0.499 | 0.479 | 0.961 | 1.613 | 1.024 | 0.194 | 0.891 | 1.814 | 1.066 | 0.207 |

Mismatch: *P*_m_ ~ *a_m_* Poisson(*λ_m_*) + (1-*a_m_*) Geometric(*p_m_*)

Insertion: *P*_i_ ~ *α_i_* Weibull(*λ_i_ , _i_*) + (1*-α_i_*) Geometric(*p_i_*)

Deletion: *P*_d_ ~ *α_d_* Weibull(*λ_d_ , _d_*) + (1*-α_d_*) Geometric(*p_d_*)


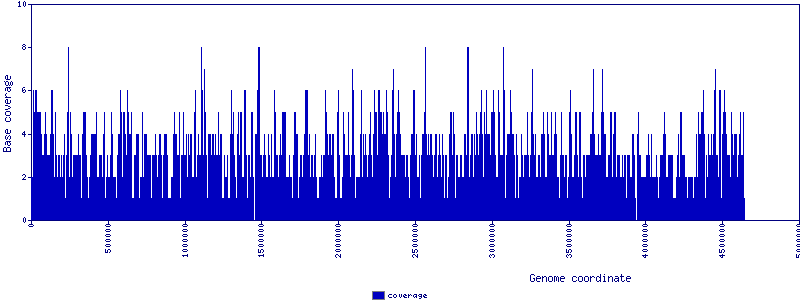


**Figure S1. *E. coli* K-12 substr. MG1655 genome coverage analysis by Full 2D (R7 chemistry) Oxford Nanopore long reads.** High-quality, Full 2D R7 nanopore reads [6] were aligned with blastn [37] onto the *E. coli* K-12 substr. MG1655 reference (U00096.2), plotting only reads with sequence identity over 50% (1,713 high quality sequences out of 3,471). We identified 184 regions 1 bp and longer with no read coverage. Overall 90.3% of the 4,639,675 bp MG1655 genome was covered by at least one nanopore read. Using a single ONT R7 run [13] provided 3,470 total full 2D reads (21,972,353 bases or 4.7-fold coverage of the *E.coli* genome). In contrast, Loman and co-workers [26] used four ONT R7.3 runs (ERX708228, ERX708229, ERX708230, ERX708231) for error correction and subsequent assembly.

**Figure S2. *E. coli* K-12 Illumina baseline assembly and genome co-linearity.** A baseline ABySS assembly (Table 1**B** in main text) of the *E. coli* K-12 MG1655 genome yields a draft genome that despite being fragmented is co-linear with the reference. Sequence comparison was performed with MUMmer v3.23 tools, using nucmer for nucleotide sequence alignments and mummerplot for plotting [38].

**Figure S3. Full 2D ONT - LINKS scaffolds co-linearity with the MG1655 genome, single *k-mer* pair LINKS run.** A single LINKS scaffolding round (k=15 bp, d=4000 bp) was performed on ABySS assembly sequence scaffolds (shown in Figure. S3B), bringing the number of scaffolds from 61 to 48 (Table 1D in manuscript) and harboring sequences in the correct order and orientation.

**Figure S4. Full 2D ONT-LINKS scaffolds co-linearity with the reference *E. coli* K-12 genome (thirty *k-*mer pair interval iterations).** Iterative LINKS scaffolding rounds (*k*=15, *d*=500 to 16000 bp, 30 iterations) were performed on ABySS assembly sequence scaffolds (Table 1F in manuscript), bringing the number of scaffolds further down to 27 from 61, with its underlying sequences in the exact configuration compared to the reference.

**Figure S5. LINKS scaffolds using all available R7 2D ONT reads compared to the reference *E. coli* K-12 genome (thirty *k-*mer pair interval iterations).** Iterative LINKS scaffolding rounds (*k*=15, *d*=500 to 16000 bp, 30 iterations) were performed on ABySS assembly sequence scaffolds (Table 1**G** in manuscript), bringing the number of scaffolds further down to 16 from 61. MUMmer co-linear analysis indicates that six large scaffolds comprise *E. coli* K-12 MG1655 re-scaffolded sequences in the correct order and orientation.

**Figure S6. LINKS scaffolds using all raw, uncorrected R7.3 ONT reads compared to the reference *E. coli* K-12 genome (thirty *k-*mer pair interval iterations).** Iterative LINKS scaffolding rounds (k=15, d=500 to 16000 bp, 30 iterations) were performed on the baseline ABySS assembly sequence scaffolds (Table 1H in manuscript), bringing the number of scaffolds down to 27 from 61. QUAST [24] analysis reveals that re-scaffolding with the raw v7.3 ONT data produces an assembly with the best compromise, with fewer errors and highest overall contiguity.


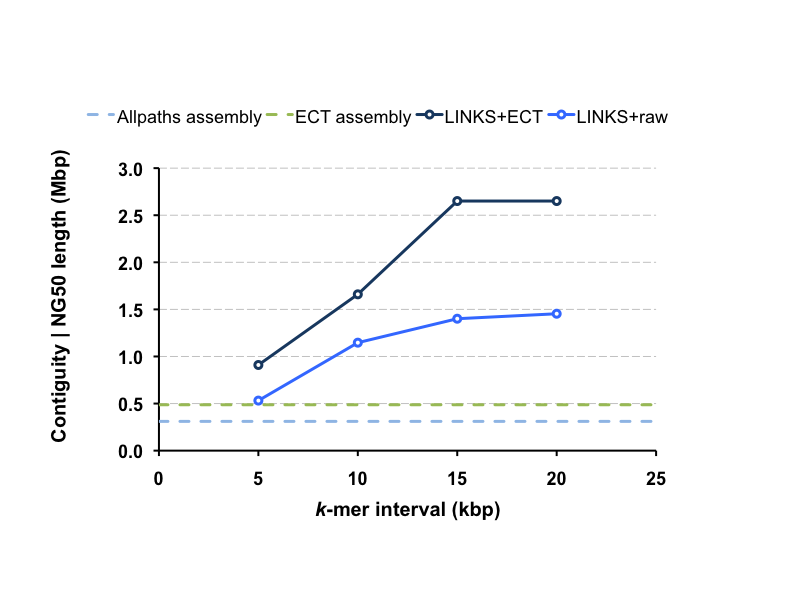


**Figure S7. LINKS re-scaffolding of a *A. thaliana* Ler-1 genome draft using raw and ECTools-corrected PacBio long reads.** We performed four rounds of iterative LINKS scaffolding of a baseline Allpaths-LG [9,29] assembly (dotted light blue line) using 5 kbp distance increment between *k-*mers (*k*=21, *t*=20|5|5|5, *l*= 5, *a*=0.3, *d*=5-20 kbp, distance step=5 kbp). The scaffolding was done using either raw (bright blue solid line) or ECTools-corrected (+ECT dark blue solid line) PacBio data [18]. We show the contiguity of the assembly, as measured by the NG50 length [23], in relation to both the baseline assembly (Baseline Allpaths-LG assembly, light blue dotted line) and an assembly of the ECTools-corrected PacBio data (ECT assembly, green dotted line).


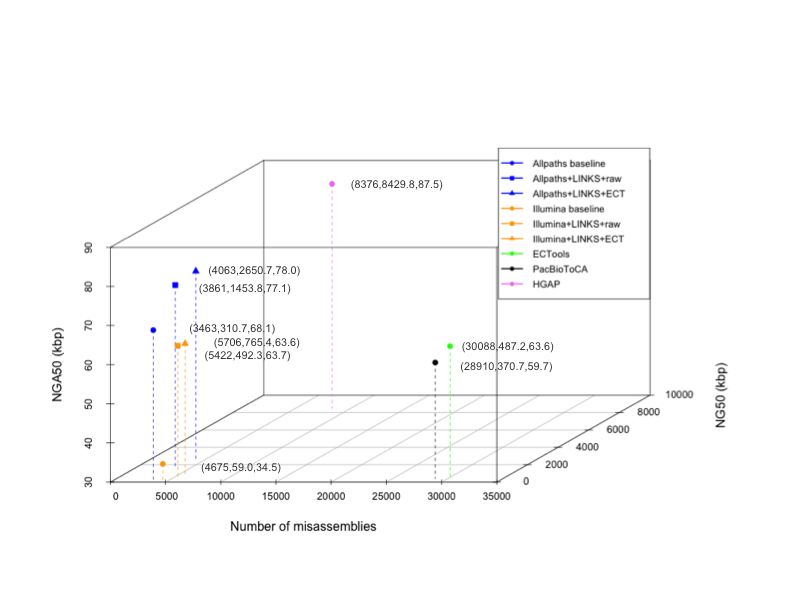


**Figure S8. LINKS assemblies of baseline *A. thaliana* Ler-1 or Ler-0 genome drafts using raw and ECTools-corrected PacBio long reads.** Final (4^th^ iteration) LINKS assemblies of baseline Allpaths-LG *A. thaliana* Ler-1 (blue symbols) or Illumina *A. thaliana* Ler-0 (orange symbols) assemblies re-scaffolded with raw (19 SMRTcells , square symbols) or ECTools (ECT)-corrected PacBio reads (19 SMRTcells, triangle symbols) were assessed by QUAST using the reference *A. thaliana* genome (GCA_000001735.1_TAIR10) and compared to other assembly strategies including ECTools (green symbol), PacBioToCA (black symbol) and HGAP (purple symbol). Whereas the HGAP assembly was more than 3x more contiguous than the Allpaths-LG assembly re-scaffolded with LINKS using ECTools corrected reads, as measured by the NG50 length metric, the corrected NGA50 metric (NG50 corrected for errors) is similar between both assemblies. The x,y,z coordinates shown in parentheses represent the number of mis-assemblies, NG50 length (kbp) and NGA50 length (kbp) in this order.

**Table S2. QUAST analysis of LINKS re-scaffolded *A. thaliana* Illumina-only assemblies compared to public assemblies of Pacific Biosciences data.**

| Assembly | Reference Genome | ECTools | PacBioToCA | HGAP | Illumina | Illumina  LINKS  raw x4 | Illumina  LINKS  ECT x4 | Allpaths-LG | Allpaths-LG LINKS  Raw x4 | Allpaths-LG LINKS  ECT x4 |
| --- | --- | --- | --- | --- | --- | --- | --- | --- | --- | --- |
| Input librairies | NA | 19 PacBio SMRTcells | 19 PacBio SMRTcells | 93 PacBio SMRTcells | Illumina MiSeq PE300, 450 bp fragment | 93 PacBio SMRTcells | 19 PacBio SMRTcells ECTools-corrected | Illumina PE101, 178 bp fragment and PE40, 2 kbp fragment | 93 PacBio SMRTcells | 19 PacBio SMRTcells, ECTools-corrected |
| Total input bases (genome fold coverage) | NA | 4.8 GB (40X, 6X over 10kbp) | 4.8 GB (40X, 6X over 10kbp) | 14.2 GB  (118X, 38X over 10kbp) | 13.8 GB (115X) | 14.2 GB (118X) | 3.4 GB  (28X) | 13.7 GB  (114X) | 14.2 GB (118X) | 3.4 GB  (28X) |
| # contigs | 5 | 74,529 | 49,545 | 1,145 | 20,530 | 17,910 | 17,039 | 1,705 | 995 | 605 |
| Largest contig | 30,427,671 | 2,029,192 | 1,621,192 | 12,431,823 | 651,509 | 2,070,278 | 4,071,260 | 2,930,102 | 4,799,970 | 6,895,571 |
| N50 | 23,459,830 | 8,341 | 9,986 | 6,100,579 | 55,598 | 436,277 | 638,133 | 341,625 | 1,524,839 | 2,766,196 |
| NG50 | 23,459,830 | 487,216 | 370,686 | 8,429,818 | 59,042 | 492,324 | 765,370 | 310,720 | 1,453,854 | 2,650,693 |
| # misassemblies | 0 | 30,088 | 28,910 | 8,376 | 4,675 | 5,422 | 5,706 | 3,463 | 3,861 | 4,063 |
| # N's per 100 kbp | 156.28 | 0.65 | 4.00 | 0.00 | 0.00 | 1,654.57 | 4,189.69 | 1,995.82 | 3,843.38 | 5,066.26 |
| Largest alignment | 30,263,548 | 718,881 | 534,469 | 724,189 | 256,783 | 722,033 | 721,884 | 715,300 | 715,300 | 715,300 |
| NA50 | 23,455,979 | 1,738 | 2,786 | 63,573 | 31,963 | 56,083 | 53,974 | 74,787 | 82,014 | 81,658 |
| NGA50 | 23,455,979 | 63,635 | 59,723 | 87,499 | 34,519 | 63,711 | 63,654 | 68,118 | 77,130 | 78,007 |

Note: LINKS, Illumina [9] and PacBio assemblies [10,18] were benchmarked against the reference *A. thaliana* GCA_000001735.1 (TAIR10). ECT: ECTools-corrected PacBio reads.

**Table S3. Read data used for LINKS scaffolding.**

| Organism | Sequencing platform | Source | Read type, chemistry | Number of reads (sequences) | Min. length (bp) | Max. Length (bp) | Mean length (bp) | N50 length (bp) | Fold  coverage |
| --- | --- | --- | --- | --- | --- | --- | --- | --- | --- |
| *E. coli* K-12 | Oxford Nanopore | http://gigadb.org/dataset/100102/ Ecoli_R7_CombinedFasta.tgz | F2D, R7 | 3,470 | 356 | 47,422 | 6,332 | 8,113 | 4.7 |
|  |  | http://gigadb.org/dataset/100102/ Ecoli_R7_CombinedFasta.tgz | 2D (F2D+Normal), R7 | 24,219 | 233 | 47,422 | 6,559 | 8,442 | 34.2 |
|  |  | https://www.ebi.ac.uk/ena/data/view/ERX708228 | Raw, R7.3 | 66,168 | 200 | 94,116 | 4,701 | 7,295 | 67.0 |
| *S.* Typhi H58 | Oxford Nanopore | http://figshare.com/articles/Salmonella_Typhi_H58_MinION_and_Illumina_data/1170110/ | 2D | 3,738 | 492 | 31,630 | 6,078 | 7,115 | 4.7 |
| *S. cerevisiae* W303 | Oxford Nanopore | http://schatzlab.cshl.edu/data/nanocorr | Raw | 249,979 | 200 | 146,992 | 5,805 | 7,949 | 119.9 |
|  |  | http://schatzlab.cshl.edu/data/nanocorr | Nanocorr | 104,787 | 200 | 72,936 | 4,657 | 8,296 | 40.3 |
| *A. thaliana* Ler-0 | Pacific Biosciences | http://schatzlab.cshl.edu/data/ectools | Raw | 3,448,228 | 35 | 41,753 | 4,137 | 7,205 | 118.9 |
|  |  | http://schatzlab.cshl.edu/data/ectools | ECTools-corrected | 288,217 | 2405 | 25,609 | 11,662 | 12,240 | 28.0 |
| *P. glauca* WS77111 | Illumina | Genbank:JZKD010000000 | Draft genome | 4,319,880 | 500 | 1,347,548 | 6,357 | 19,894 | ~1.2 |

*F2D: Full 2D reads, 2D: 2D reads, ECTools-corrected: ECTools-corrected PacBio reads.

**Table S4. Baseline assemblies used for scaffolding.**

| Organism | Genome Size (Mbp) | Data origin | Source |
| --- | --- | --- | --- |
| *E. coli* K-12 MG1655 | 4.6 | Illumina | Illumina BaseSpace, re-sampled to 241x coverage before ABySS v1.5.2 assembly |
| *S.* Typhi haplotype H58 | 4.8 | Illumina | Genbank:GCA_000944835.1 |
| *S. cerevisiae* W303 | 11.8 | Illumina | http://schatzlab.cshl.edu/data/nanocorr |
| *S. cerevisiae* S288c | 12.1 | Illumina | https://www.ebi.ac.uk/ena/data/view/ERR156523, ABySS v1.5.2 assembly |
| *A. thaliana* | 119.1 | Illumina | http://1001genomes.org/data/MPI/MPISchneeberger2011/releases/current/Ler-1/Assemblies/Allpaths_LG/ |
|  |  | Illumina | http://schatzlab.cshl.edu/data/ectools |
| *P. glauca* PG29 | 2078.0 | Illumina | Genbank:ALWZ030000000 |

**Supplementary references**

37. Altschul SF, Gish W, Miller W, Myers EW, Lipman DJ. Basic local alignment search tool. J Mol Biol. 1990;215:403-10.

38. Kurtz S, Phillippy A, Delcher AL, Smoot M, Shumway M, Antonescu C, Salzberg SL. Versatile and open software for comparing large genomes. Genome Biol. 2004;5:R12.
